# Syntheses and crystal structures of (*R*,*R*)- and (*S*,*S*)-bis­(aceto­nitrile-κ*N*)[*N*,*N*′-dimethyl-*N*,*N*′-bis(pyridin-2-ylmeth­yl)cyclo­hexane-1,2-di­amine-κ^4^*N*]iron(II) bis­(hexa­fluoro­anti­monate)

**DOI:** 10.1107/S2056989024011307

**Published:** 2024-11-28

**Authors:** Yurii S. Bibik, Iryna M. Yulakh, Svitlana V. Shishkina, Dmytro M. Khomenko, Roman O. Doroshchuk, Ilona V. Raspertova, Rostyslav D. Lampeka

**Affiliations:** a Department of Chemistry Kyiv National Taras Shevchenko University Volodymyrska st 64 Kyiv Ukraine; b Enamine Ltd. (www.enamine.net), Winston Churchill St.78, Kyiv 02094, Ukraine; c SSI "Institute for Single Crystals", National Academy of Sciences of Ukraine, Nauky ave. 60, 61001 Kharkiv, Ukraine; d Institute of Organic Chemistry, National Academy of Sciences of Ukraine, Akademika Kukharya Street 5, 02098 Kyiv, Ukraine; University of Aberdeen, United Kingdom

**Keywords:** crystal structure, X-ray crystallography, non-heme iron catalysts

## Abstract

Two enanti­omeric non-heme iron complexes [Fe(*R*,*R*-BPMCN)(CH_3_CN)_2_](SbF_6_)_2_ and [Fe(*S*,*S*-BPMCN)(CH_3_CN)_2_](SbF_6_)_2_ (BPMCM = *N*,*N*′-dimethyl-*N*,*N*′-bis­(pyridin-2-ylmeth­yl)-cyclo­hexane-1,2-di­amine, C_22_H_28_N_4_) were obtained in parallel syntheses starting from the enanti­omerically pure *R*,*R* and *S*,*S* BPMCN ligands. The Fe^II^ cations have a distorted octa­hedral FeN_6_ geometry formed by a chelating *N*,*N*,*N*,*N*-tetra­dentate BPMCN ligand and two mol­ecules of aceto­nitrile. The ligand adopts a *cis*-*α* topology with the two pyridine groups coordinated *trans* to each other. In the crystals, a system of C—H⋯F hydrogen bonds links the cations to the hexa­fluoro­anti­monate anions, resulting in a three-dimensional architecture.

## Abstract 2

The syntheses and crystal structures of two enanti­omeric non-heme iron catalysts based on *N*,*N*′-dimethyl-*N*,*N*′-bis­(pyridin-2-ylmeth­yl)cyclo­hexane-1,2-di­amine are described.

## 1. Chemical context

Non-heme iron complexes show promising stereospecific hy­droxy­lation reactivity with unactivated *sp*^3^ C—H bonds in various hydro­carbon substrates (Gormisky & White, 2013 ▸; White *et al.*, 2001 ▸; Zhang & Goldsmith, 2014 ▸; Chen & White, 2007 ▸; Chen *et al.*, 2018 ▸; Esarey *et al.*, 2016 ▸; Gómez *et al.*, 2009 ▸, 2013 ▸; Font *et al.*, 2016 ▸; Siedlecka, 2023 ▸). However, their use in preparative C—H oxidation chemistry has been limited by the need for a large excess of substrate relative to the oxidant, low catalyst turnover numbers, and poor selectivity in product formation. Despite these challenges, iron complexes with *N*,*N*′-dimethyl-*N*,*N*′-bis­(pyridin-2-ylmeth­yl)-ethane-1,2-di­amine show potential for preparative C—H oxidations with complex substrates due to their operation *via* an electrophilic metal oxidant (Chen & Que, 1999 ▸; Okuno *et al.*, 1997 ▸), their bulky, modifiable ligand framework, and their successful use in preparative epoxidations of functionalized olefins (White *et al.*, 2001 ▸). Exchanging the ethyl­ene bridge with a cyclo­hexane ring is one of the ways of modifying the structure of the complex without losing the rigidity of the framework, which is important for catalytic activity (Zhang & Goldsmith, 2014 ▸; Chen & White, 2007 ▸; Esarey *et al.*, 2016 ▸; Gómez *et al.*, 2009 ▸, 2013 ▸; Font *et al.*, 2016 ▸; Costas, Tipton *et al.*, 2001 ▸). Considering the above, we now report the synthesis and crystal structures of two enanti­omeric analogues Fe(*R*,*R*-BPMCN)(CH_3_CN)_2_(SbF_6_)_2_ (I) and Fe(*S*,*S*-BPMCN)(CH_3_CN)_2_(SbF_6_)_2_ (II) of the White–Chen catalyst based on *N*,*N*′-dimethyl-*N*,*N*′-bis­(pyridin-2-ylmeth­yl)-cyclo­hexane-1,2-di­amine (BPMCN; C_22_H_28_N_4_).
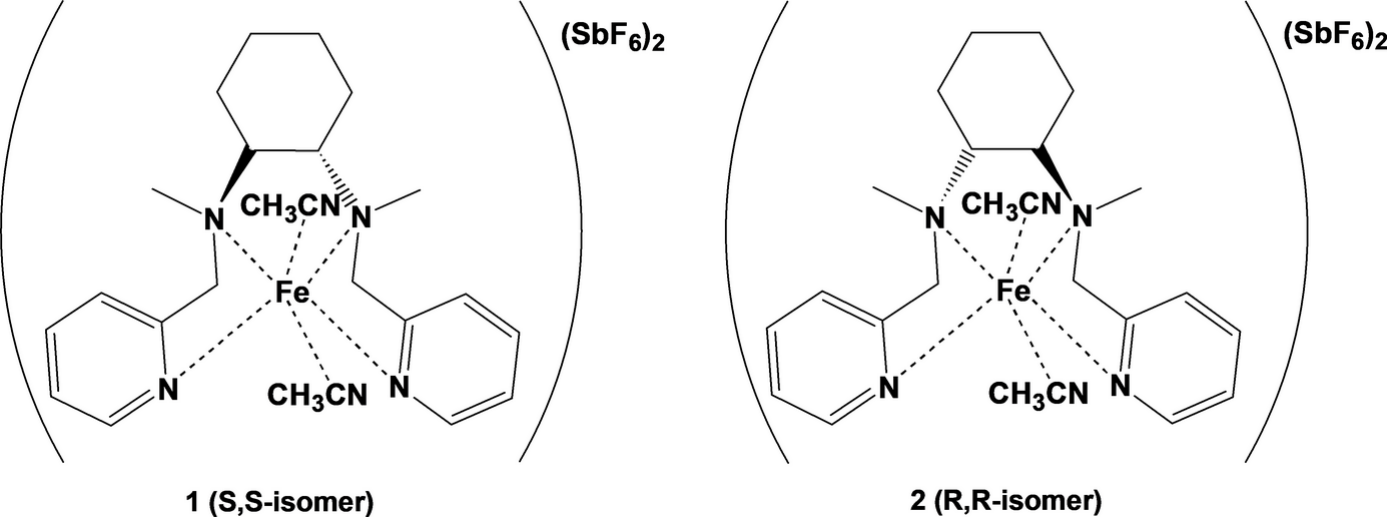


## 2. Structural commentary

**Figure 1:**
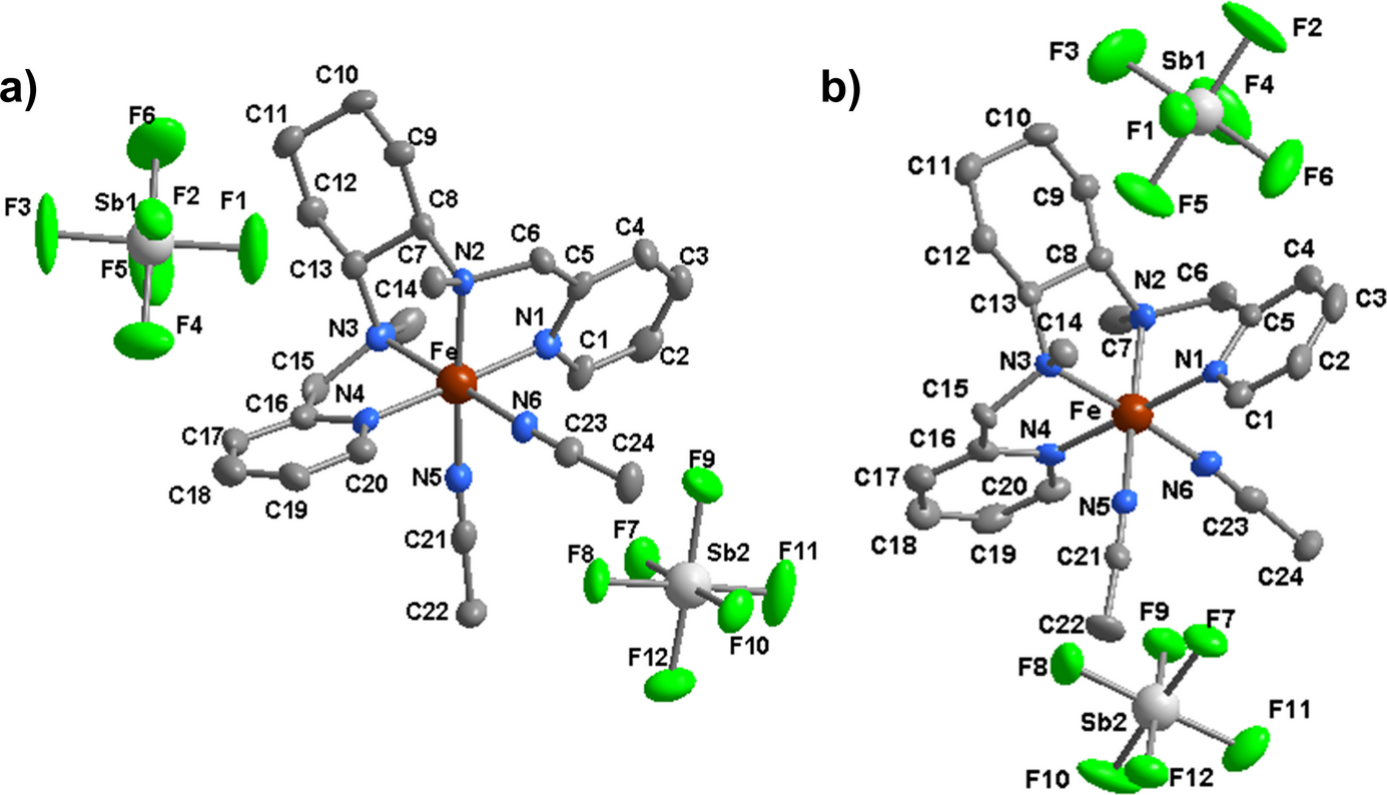
The mol­ecular structures of the title compounds (*a*) (I) and (*b*) (II) with displacement ellipsoids shown at the 50% level.

**Figure 2:**
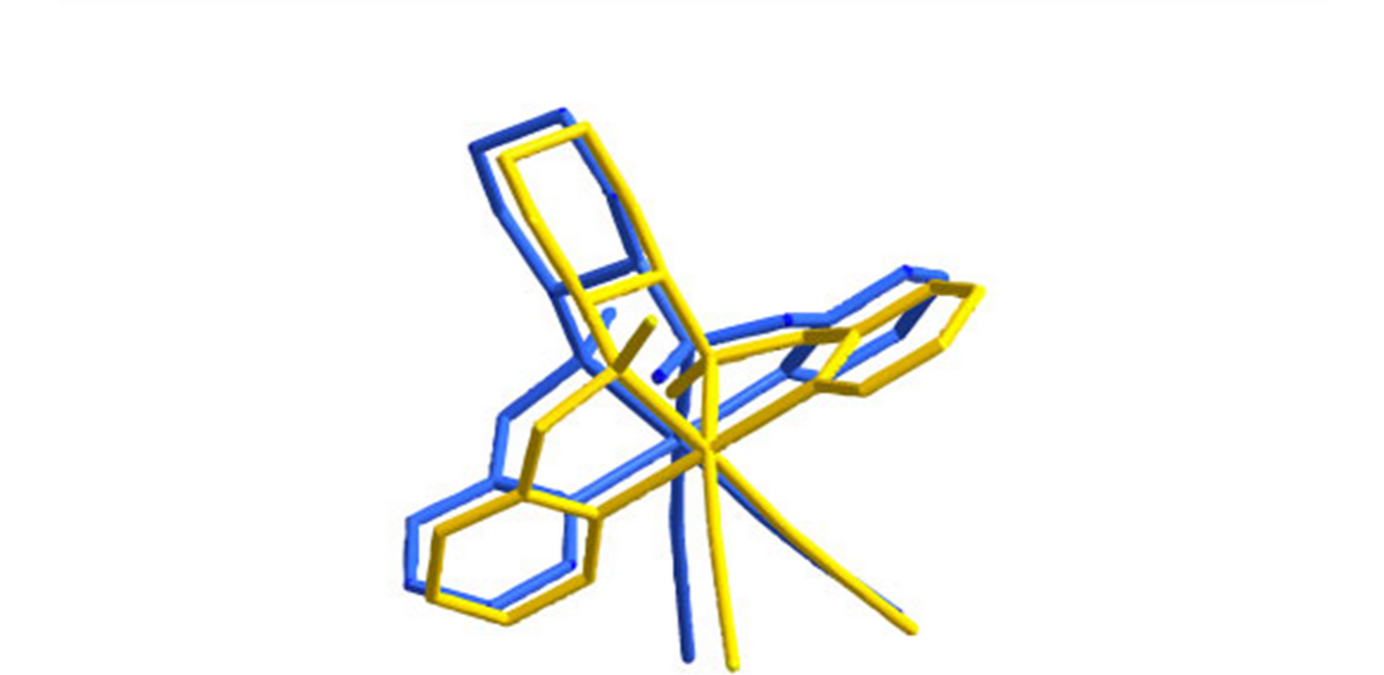
Overlay of the mol­ecular structures of the cations of (I) (blue) and (II) (yellow) showing the difference in the absolute structures.

Single crystals of both enanti­omers were obtained *via* gas diffusion in an MTBE/aceto­nitrile solvent system. Both structures are built up from [Fe(BPMCN)(CH_3_CN)_2_]^2+^ complex cations and hexa­fluoro­anti­monate anions in a 1:2 ratio. They crystallize in the ortho­rhom­bic Sohncke space group *P*2_1_2_1_2_1_ with four formula units per unit cell (Fig. 1 ▸). Each iron(II) ion has an N_6_ coordination environment in a distorted octa­hedral geometry provided by a chelating tetra­dentate BPMCN ligand and two mol­ecules of aceto­nitrile in adjacent (*cis*) positions. The structures reveal that the ligand adopts a *cis*-*α* topology in which the two pyridine groups coordinate *trans* to each other and the two N—Me groups are oriented *anti* to each other. In (I), the stereogenic centres C8 and C13 have *R* configurations and in (II), the equivalent atoms have *S* configurations. The values of bond distances are given in Tables 1 ▸ and 2 ▸. The average Fe–N distances are 1.99 and 1.98 Å for (I) and (II), respectively, which is consistent with a low spin 3*d*^6^ state for Fe^2+^. The pyridine rings are rotated relative to each other by 62.8 (6)° for (I) and 63.4 (5)° for (II). The structures of the complex ions in (I) and (II) are compared in Fig. 2 ▸, where the opposite orientations of the N—Me groups can clearly be seen.

## 3. Supra­molecular features

**Figure 3:**
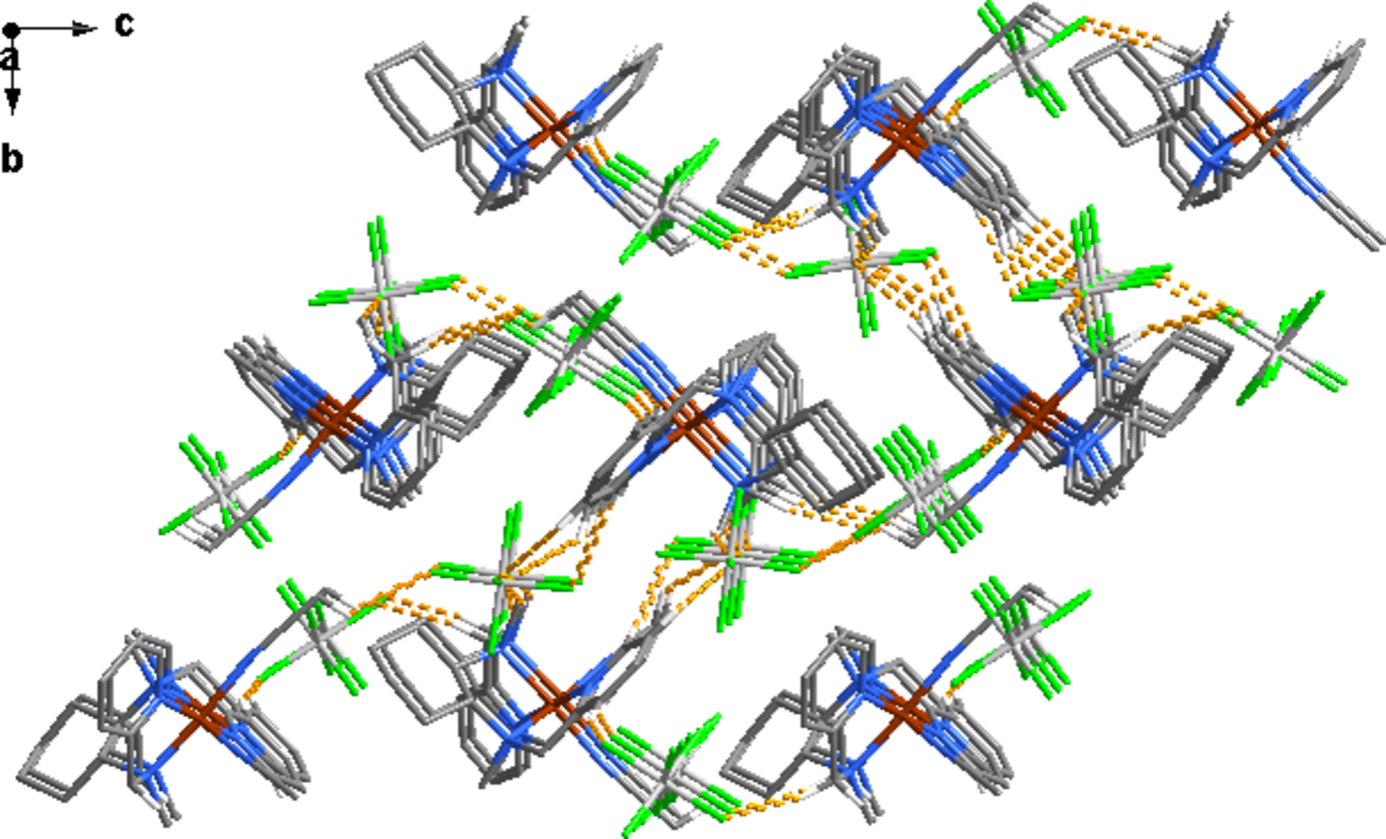
The crystal structure of (I) viewed along the *a*-axis direction.

In the crystals, the complex cations form chains propagating along the *a*-axis direction (Fig. 3 ▸). Within and outside the chains, the cations are inter­connected *via* C—H⋯F hydrogen bond contacts with the hexa­fluoro­anti­monate anions that fill the space between the complex ions (Tables 3 ▸ and 4 ▸).

## 4. Database survey

A search of the Cambridge Structural Database (CSD, version 5.43, updated March 2022; (Groom *et al.*, 2016 ▸)) revealed 187 hits for transition metal complexes containing a ligand with an *N*,*N*′-dimethyl-cyclo­hexane-1,2-di­amine fragment; 30 of which include iron (II or III). Additionally, there are 28 hits for complexes with the title ligand and various co-ligands (*e.g.*, chloride or triflate anions, amino acids, solvents, among others). Notably, complexes have been found with metals such as manganese(II) (CSD refcode BASGAE; Murphy *et al.*, 2003 ▸, HEWJOI; Glerup *et al.*, 1994 ▸), nickel(II) (MANYOS, (Wang *et al.*, 2016 ▸), cobalt(III) (LUXGIU; Kooistra *et al.*, 2003 ▸); IGEQOA; Leverett *et al.*, 1999 ▸); NEFRAR; Leverett *et al.*, 1996 ▸); VIVREX; Fenton *et al.*, 1991 ▸); ZOXGIC; Fenton *et al.*, 1995 ▸), zinc(II) (KEWHEA; Kim *et al.*, 2006 ▸), ruthenium(II, III) (CEVSII; Tse *et al.*, 2018 ▸), XULVAB; Aldrich-Wright *et al.*, 2002 ▸), rhenium(V, VI) (GESREE, GESRII, GESRUU; Ng *et al.*, 2017 ▸), osmium(III, V) (FOGNUN, FOGTAZ, FOGTED, FOGTIH, FOGTON, FOGTUT; Fujimoto *et al.*, 2019 ▸).

Furthermore, the following closely related iron analogues are noteworthy: Fe(BPMCN)(OTf)_2_ and Fe(6-Me_2_-BPMCN)(OTf)_2_ (UBOWEN and UBOWIR; Costas, Tipton *et al.*, 2001 ▸), and Fe(5-Me_2_-BPMCN)(OTf)_2_ (ODECIJ; Costas, Rohde *et al.*, 2001 ▸). These structures crystallize in the monoclinic crystal system. Notably, in the latter two structures, which contain a methyl group as a substituent, the ligand adopts a *cis*-*β* topology where the two pyridine groups coordinate in a *cis* arrangement. Additionally, a dimeric compound was identified: Fe_2_(BPMCN)_2_(OH)_2_(OTf)_2_ (FAVPAU; Stubna *et al.*, 2004 ▸), which crystallizes in a triclinic space group and exhibits a *cis*-*α* topology similar to that of the title compounds.

## 5. Synthesis and crystallization

The chiral *R*,*R-*BPMCN ligand (483 mg, 1.5 mmol, 1.0 equiv.) was dissolved in 5 ml of aceto­nitrile in a 10 ml round-bottom flask. The flask was then filled with argon, and FeCl_2_·4H_2_O (297 mg, 1.5 mmol, 1.0 equiv.) was added. The reaction mixture stirred for 4 h at room temperature. Immediately after adding the iron salt, the formation of an orange precipitate was observed. After stirring, a few drops of methyl *tert*-butyl ether (MTBE) were added to the mixture to fully precipitate the product, and the mixture was filtered. The precipitate was washed with aceto­nitrile (3 × 3 ml) and MTBE (3 ml). Yield: 342 mg (0.76 mmol, 51%) of Fe(*R*,*R*-BPMCN)Cl_2_ as a bright-orange power. The reaction was repeated for the *S*,*S-*BPMCN ligand to yield 270 mg (0.60 mmol, 40%) of Fe(*S*,*S*-BPMCN)Cl_2_ as a bright-orange powder.

Fe(*R*,*R*-BPMCN)Cl_2_ (226 mg, 0.5 mmol, 1.0 equiv.) was dispersed in 10 ml of aceto­nitrile in a 25 ml flask. The flask was then filled with argon, and AgSbF_6_ (344 mg, 1.0 mmol, 2.0 equiv.) was added, which immediately caused the precipitation of AgCl and a color change of the solution to dark red. The reaction flask was wrapped in foil to protect it from light and stirred vigorously for 4 h. The reaction mixture was filtered and concentrated almost to dryness, then redissolved in aceto­nitrile and filtered again. The filtration process was repeated three times to ensure complete removal of silver salts. The filtrate was then evaporated, yielding the product (I) as a light red powder, yield 436 mg (0.467 mmol, 93%). The process was repeated starting from Fe(*S*,*S*-BPMCN)Cl_2_, resulting in 442 mg (0.473 mmol, 94%) of (II).

Crystals of (I) and (II) suitable for X-ray structural analysis were obtained by dissolving 5 mg of the complex in a minimum of aceto­nitrile (0.1 m). The loosely sealed vial with the solution was placed in a larger vial containing 1 ml of MTBE for 24 h to grow fine single crystals via gas diffusion.

## 6. Refinement

Crystal data, data collection and structure refinement details are summarized in Table 5 ▸. All hydrogen atoms were placed geometrically and refined as riding atoms, with C—H = 0.98 Å (CH_2_), 0.99 Å (CH_3_) or 0.95 Å (C_arom_), and with *U*_iso_(H) = 1.2*U*_eq_(C_arom_) or 1.5*U*_eq_(C_aliph_).

## Supplementary Material

Crystal structure: contains datablock(s) global, II, I. DOI: 10.1107/S2056989024011307/hb8113sup1.cif

Structure factors: contains datablock(s) I. DOI: 10.1107/S2056989024011307/hb8113Isup4.hkl

Structure factors: contains datablock(s) II. DOI: 10.1107/S2056989024011307/hb8113IIsup5.hkl

CCDC references: 2404184, 2404183

Additional supporting information:  crystallographic information; 3D view; checkCIF report

## Figures and Tables

**Table 1:** Selected bond lengths (Å) for (I)

Fe1—N1	1.995 (9)	Fe1—N4	1.985 (9)
Fe1—N2	2.042 (8)	Fe1—N5	1.942 (9)
Fe1—N3	2.031 (9)	Fe1—N6	1.950 (9)

**Table 2:** Selected bond lengths (Å) for (II)

Fe1—N1	1.983 (7)	Fe1—N4	1.985 (7)
Fe1—N2	2.024 (7)	Fe1—N5	1.942 (7)
Fe1—N3	2.031 (7)	Fe1—N6	1.936 (7)

**Table 3:** Hydrogen-bond geometry (Å, °) for (I)

*D*—H⋯*A*	*D*—H	H⋯*A*	*D*⋯*A*	*D*—H⋯*A*
C6—H6*A*⋯F3^i^	0.99	2.45	3.141 (13)	127
C6—H6*B*⋯F12^ii^	0.99	2.38	3.302 (13)	154
C7—H7*A*⋯F1	0.98	2.39	3.307 (13)	157
C17—H17⋯F9^iii^	0.95	2.41	3.260 (15)	149
C19—H19⋯F3^iv^	0.95	2.52	3.372 (17)	150
C20—H20⋯F4^iv^	0.95	2.54	3.385 (15)	148
C22—H22*A*⋯F6^v^	0.98	2.51	3.113 (16)	120
C24—H24*B*⋯F1^iv^	0.98	2.44	3.232 (16)	138

Symmetry codes: (i) 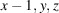; (ii) 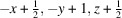; (iii) 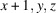; (iv) 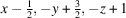; (v) 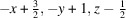.

**Table 4:** Hydrogen-bond geometry (Å, °) for (II)

*D*—H⋯*A*	*D*—H	H⋯*A*	*D*⋯*A*	*D*—H⋯*A*
C1—H1⋯F6^i^	0.95	2.54	3.385 (12)	148
C2—H2⋯F2^ii^	0.95	2.52	3.371 (15)	149
C4—H4⋯F8^iii^	0.95	2.40	3.246 (13)	148
C14—H14*C*⋯F5	0.98	2.41	3.317 (10)	155
C15—H15*A*⋯F11^iv^	0.99	2.40	3.322 (11)	154
C15—H15*B*⋯F2^v^	0.99	2.46	3.149 (11)	126
C22—H22*B*⋯F5^i^	0.98	2.50	3.241 (12)	133
C24—H24*B*⋯F3^vi^	0.98	2.47	3.098 (12)	122

Symmetry codes: (i) 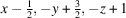; (ii) 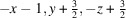; (iii) 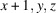; (iv) 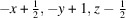; (v) 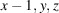; (vi) 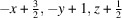.

**Table 5:** Experimental details

	(I)	(II)
Crystal data		
Chemical formula	[Fe(C_22_H_28_N_4_)(C_2_H_3_N)_2_](SbF_6_)_2_	[Fe(C_22_H_28_N_4_)(C_2_H_3_N)_2_](SbF_6_)_2_
*M*_r_	933.92	933.92
Crystal system, space group	Orthorhombic, *P*2_1_2_1_2_1_	Orthorhombic, *P*2_1_2_1_2_1_
Temperature (K)	173	173
*a*, *b*, *c* (Å)	11.4949 (5), 15.1609 (7), 18.5616 (9)	11.4986 (4), 15.1454 (4), 18.5733 (5)
*V* (Å^3^)	3234.8 (3)	3234.56 (17)
*Z*	4	4
Radiation type	Mo *K*α	Mo *K*α
μ (mm^−1^)	2.20	2.20
Crystal size (mm)	0.2 × 0.1 × 0.05	0.15 × 0.13 × 0.08

Data collection		
Diffractometer	Bruker APEXII CCD	Bruker APEXII CCD
Absorption correction	Multi-scan (*SADABS*; Krause *et al.*, 2015 ▸)	Multi-scan (*SADABS*; Krause *et al.*, 2015 ▸)
*T*_min_, *T*_max_	0.481, 0.746	0.582, 0.746
No. of measured, independent and observed [*I* > 2σ(*I*)] reflections	52192, 7430, 5313	40783, 9446, 6490
*R*_int_	0.145	0.095
(sin θ/λ)_max_ (Å^−1^)	0.650	0.703

Refinement		
*R*[*F*^2^ > 2σ(*F*^2^)], *wR*(*F*^2^), *S*	0.058, 0.120, 1.02	0.064, 0.110, 1.05
No. of reflections	7430	9446
No. of parameters	410	410
H-atom treatment	H-atom parameters constrained	H-atom parameters constrained
Δρ_max_, Δρ_min_ (e Å^−3^)	1.23, −0.57	1.36, −1.25
Absolute structure	Flack *x* determined using 1767 quotients [(*I*^+^)−(*I*^−^)]/[(*I*^+^)+(*I*^−^)] (Parsons *et al.*, 2013 ▸)	Flack *x* determined using 2001 quotients [(*I*^+^)−(*I*^−^)]/[(*I*^+^)+(*I*^−^)] (Parsons *et al.*, 2013 ▸)
Absolute structure parameter	−0.02 (2)	−0.021 (17)

Computer programs: *APEX2* and *SAINT* (Bruker, 2014 ▸), *SHELXT2014/5* (Sheldrick, 2015*a* ▸), *SHELXL2016/6* (Sheldrick, 2015*b* ▸) and *OLEX2* (Dolomanov *et al.*, 2009 ▸).

## supplementary crystallographic information

### (S,S)-Bis(acetonitrile-κN)[N,N'-dimethyl-N,N'-bis(pyridin-2-ylmethyl)cyclohexane-1,2-diamine-κ4N]iron(II) bis(hexafluoroantimonate) (I) Crystal data

**Table:**

[Fe(C_22_H_28_N_4_)(C_2_H_3_N)_2_](SbF_6_)_2_	*D*_x_ = 1.918 Mg m^−^^3^
*M**_r_* = 933.92	Mo *K*α radiation, λ = 0.71073 Å
Orthorhombic, *P*2_1_2_1_2_1_	Cell parameters from 3614 reflections
*a* = 11.4949 (5) Å	θ = 2.2–17.6°
*b* = 15.1609 (7) Å	µ = 2.20 mm^−^^1^
*c* = 18.5616 (9) Å	*T* = 173 K
*V* = 3234.8 (3) Å^3^	Plate, red
*Z* = 4	0.2 × 0.1 × 0.05 mm
*F*(000) = 1824	

### (S,S)-Bis(acetonitrile-κN)[N,N'-dimethyl-N,N'-bis(pyridin-2-ylmethyl)cyclohexane-1,2-diamine-κ4N]iron(II) bis(hexafluoroantimonate) (I) Data collection

**Table:**

Bruker APEXII CCD diffractometer	5313 reflections with *I* > 2σ(*I*)
φ and ω scans	*R*_int_ = 0.145
Absorption correction: multi-scan (SADABS; Krause *et al.*, 2015)	θ_max_ = 27.5°, θ_min_ = 2.1°
*T*_min_ = 0.481, *T*_max_ = 0.746	*h* = −14→14
52192 measured reflections	*k* = −19→19
7430 independent reflections	*l* = −24→23

### (S,S)-Bis(acetonitrile-κN)[N,N'-dimethyl-N,N'-bis(pyridin-2-ylmethyl)cyclohexane-1,2-diamine-κ4N]iron(II) bis(hexafluoroantimonate) (I) Refinement

**Table:**

Refinement on *F*^2^	Hydrogen site location: inferred from neighbouring sites
Least-squares matrix: full	H-atom parameters constrained
*R*[*F*^2^ > 2σ(*F*^2^)] = 0.058	*w* = 1/[σ^2^(*F*_o_^2^) + (0.0477*P*)^2^ + 0.5546*P*] where *P* = (*F*_o_^2^ + 2*F*_c_^2^)/3
*wR*(*F*^2^) = 0.120	(Δ/σ)_max_ < 0.001
*S* = 1.01	Δρ_max_ = 1.23 e Å^−^^3^
7430 reflections	Δρ_min_ = −0.56 e Å^−^^3^
410 parameters	Absolute structure: Flack *x* determined using 1767 quotients [(*I*^+^)-(*I*^-^)]/[(*I*^+^)+(*I*^-^)] (Parsons *et al.*, 2013)
0 restraints	Absolute structure parameter: −0.02 (2)
Primary atom site location: dual	

### (S,S)-Bis(acetonitrile-κN)[N,N'-dimethyl-N,N'-bis(pyridin-2-ylmethyl)cyclohexane-1,2-diamine-κ4N]iron(II) bis(hexafluoroantimonate) (I) Special details

**Table:**

Geometry. All esds (except the esd in the dihedral angle between two l.s. planes) are estimated using the full covariance matrix. The cell esds are taken into account individually in the estimation of esds in distances, angles and torsion angles; correlations between esds in cell parameters are only used when they are defined by crystal symmetry. An approximate (isotropic) treatment of cell esds is used for estimating esds involving l.s. planes.

### (S,S)-Bis(acetonitrile-κN)[N,N'-dimethyl-N,N'-bis(pyridin-2-ylmethyl)cyclohexane-1,2-diamine-κ4N]iron(II) bis(hexafluoroantimonate) (I) Fractional atomic coordinates and isotropic or equivalent isotropic displacement parameters (Å^2^)

**Table:**

	*x*	*y*	*z*	*U*_iso_*/*U*_eq_	
Sb1	1.02115 (7)	0.72812 (6)	0.66575 (5)	0.0433 (2)	
F1	0.8598 (6)	0.7251 (6)	0.6632 (6)	0.091 (3)	
F2	1.0176 (6)	0.8502 (4)	0.6516 (4)	0.0538 (19)	
F3	1.1834 (6)	0.7345 (7)	0.6700 (7)	0.110 (4)	
F4	1.0271 (9)	0.7172 (6)	0.5668 (5)	0.093 (3)	
F5	1.0183 (8)	0.6080 (6)	0.6806 (7)	0.118 (4)	
F6	1.0155 (10)	0.7467 (8)	0.7625 (5)	0.117 (4)	
Sb2	0.16563 (6)	0.38096 (6)	0.43454 (5)	0.0411 (2)	
F7	0.2382 (6)	0.2908 (5)	0.4880 (4)	0.059 (2)	
F8	0.3088 (5)	0.4370 (5)	0.4185 (4)	0.053 (2)	
F9	0.1472 (7)	0.4470 (7)	0.5181 (5)	0.082 (3)	
F10	0.0909 (6)	0.4696 (5)	0.3815 (4)	0.053 (2)	
F11	0.0226 (7)	0.3246 (6)	0.4465 (5)	0.089 (3)	
F12	0.1896 (8)	0.3167 (5)	0.3508 (4)	0.063 (2)	
Fe1	0.53506 (12)	0.50721 (9)	0.59732 (8)	0.0255 (3)	
N1	0.3957 (7)	0.4683 (5)	0.6529 (5)	0.028 (2)	
N2	0.5315 (6)	0.6063 (5)	0.6717 (4)	0.0230 (17)	
N3	0.6562 (8)	0.4500 (5)	0.6618 (5)	0.031 (2)	
N4	0.6763 (7)	0.5451 (6)	0.5442 (5)	0.031 (2)	
N5	0.5283 (8)	0.4080 (6)	0.5313 (5)	0.034 (2)	
N6	0.4278 (7)	0.5706 (6)	0.5345 (5)	0.028 (2)	
C1	0.3416 (8)	0.3894 (7)	0.6490 (6)	0.032 (2)	
H1	0.374334	0.344605	0.619448	0.038*	
C2	0.2409 (10)	0.3717 (8)	0.6862 (6)	0.041 (3)	
H2	0.204958	0.315477	0.682080	0.049*	
C3	0.1913 (9)	0.4367 (8)	0.7302 (7)	0.040 (3)	
H3	0.122741	0.425280	0.757136	0.048*	
C4	0.2457 (9)	0.5183 (8)	0.7331 (6)	0.034 (3)	
H4	0.213776	0.564454	0.761540	0.041*	
C5	0.3470 (9)	0.5321 (7)	0.6940 (6)	0.027 (2)	
C6	0.4077 (8)	0.6192 (7)	0.6918 (6)	0.028 (2)	
H6A	0.369421	0.658092	0.656122	0.033*	
H6B	0.402824	0.647898	0.739600	0.033*	
C7	0.5773 (9)	0.6911 (7)	0.6440 (6)	0.030 (3)	
H7A	0.662037	0.687320	0.639399	0.046*	
H7B	0.557122	0.738588	0.677590	0.046*	
H7C	0.542885	0.703402	0.596783	0.046*	
C8	0.5959 (8)	0.5750 (7)	0.7381 (6)	0.026 (2)	
H8	0.541977	0.535367	0.765333	0.031*	
C9	0.6351 (9)	0.6475 (7)	0.7899 (6)	0.031 (3)	
H9A	0.686756	0.689104	0.764162	0.038*	
H9B	0.566229	0.680767	0.806920	0.038*	
C10	0.6991 (10)	0.6095 (8)	0.8542 (6)	0.040 (3)	
H10A	0.647049	0.569359	0.881259	0.047*	
H10B	0.723518	0.657646	0.886874	0.047*	
C11	0.8075 (9)	0.5581 (8)	0.8273 (7)	0.040 (3)	
H11A	0.859723	0.598315	0.800486	0.048*	
H11B	0.850974	0.534018	0.868925	0.048*	
C12	0.7686 (9)	0.4829 (8)	0.7783 (6)	0.035 (3)	
H12A	0.719277	0.441412	0.805906	0.042*	
H12B	0.837570	0.450274	0.760780	0.042*	
C13	0.6996 (8)	0.5188 (7)	0.7137 (6)	0.027 (2)	
H13	0.753059	0.558508	0.686178	0.033*	
C14	0.6159 (10)	0.3691 (7)	0.6990 (7)	0.042 (3)	
H14A	0.561986	0.385185	0.737746	0.063*	
H14B	0.682935	0.337951	0.719452	0.063*	
H14C	0.576155	0.330646	0.664452	0.063*	
C15	0.7553 (10)	0.4274 (8)	0.6131 (7)	0.045 (3)	
H15A	0.827133	0.419239	0.641775	0.053*	
H15B	0.738591	0.371514	0.587525	0.053*	
C16	0.7723 (9)	0.4987 (8)	0.5606 (6)	0.038 (3)	
C17	0.8793 (10)	0.5165 (10)	0.5279 (7)	0.049 (4)	
H17	0.946372	0.483421	0.540693	0.059*	
C18	0.8868 (12)	0.5829 (10)	0.4767 (8)	0.054 (4)	
H18	0.959053	0.596953	0.454693	0.065*	
C19	0.7858 (10)	0.6284 (9)	0.4582 (6)	0.045 (3)	
H19	0.787372	0.672452	0.421875	0.054*	
C20	0.6828 (10)	0.6084 (8)	0.4938 (6)	0.034 (3)	
H20	0.614530	0.640657	0.482044	0.041*	
C21	0.5172 (9)	0.3580 (7)	0.4853 (6)	0.034 (3)	
C22	0.5019 (9)	0.2955 (7)	0.4263 (7)	0.041 (3)	
H22A	0.475302	0.327063	0.383210	0.062*	
H22B	0.443818	0.251247	0.439942	0.062*	
H22C	0.576091	0.266340	0.416037	0.062*	
C23	0.3584 (9)	0.6009 (7)	0.5003 (6)	0.029 (3)	
C24	0.2624 (10)	0.6404 (8)	0.4577 (7)	0.044 (3)	
H24A	0.248725	0.604767	0.414480	0.066*	
H24B	0.283730	0.700561	0.443489	0.066*	
H24C	0.191489	0.642060	0.486932	0.066*	

### (S,S)-Bis(acetonitrile-κN)[N,N'-dimethyl-N,N'-bis(pyridin-2-ylmethyl)cyclohexane-1,2-diamine-κ4N]iron(II) bis(hexafluoroantimonate) (I) Atomic displacement parameters (Å^2^)

**Table:**

	*U*^11^	*U*^22^	*U*^33^	*U*^12^	*U*^13^	*U*^23^
Sb1	0.0265 (4)	0.0534 (5)	0.0499 (5)	0.0049 (4)	0.0024 (4)	0.0139 (4)
F1	0.030 (4)	0.101 (7)	0.141 (9)	−0.009 (4)	−0.002 (5)	0.056 (7)
F2	0.053 (4)	0.043 (4)	0.066 (5)	0.002 (3)	0.008 (4)	−0.004 (3)
F3	0.018 (4)	0.116 (8)	0.198 (11)	0.007 (4)	0.003 (5)	0.058 (8)
F4	0.125 (8)	0.095 (7)	0.060 (6)	−0.053 (6)	0.019 (6)	−0.023 (5)
F5	0.073 (6)	0.059 (6)	0.222 (13)	0.014 (5)	0.023 (8)	0.061 (7)
F6	0.120 (8)	0.182 (11)	0.051 (6)	−0.033 (8)	−0.013 (6)	0.043 (7)
Sb2	0.0295 (4)	0.0507 (5)	0.0430 (5)	0.0013 (4)	−0.0087 (4)	−0.0013 (4)
F7	0.044 (4)	0.081 (6)	0.053 (5)	0.009 (4)	−0.008 (4)	0.024 (4)
F8	0.023 (3)	0.054 (4)	0.083 (6)	−0.006 (3)	−0.004 (3)	−0.009 (4)
F9	0.058 (5)	0.135 (8)	0.054 (6)	0.036 (5)	0.000 (4)	−0.028 (5)
F10	0.045 (4)	0.049 (5)	0.065 (6)	0.005 (3)	−0.020 (4)	0.009 (4)
F11	0.037 (4)	0.119 (7)	0.112 (8)	−0.020 (5)	−0.022 (5)	0.071 (6)
F12	0.092 (6)	0.048 (5)	0.049 (5)	−0.005 (4)	−0.016 (4)	−0.010 (4)
Fe1	0.0220 (7)	0.0260 (8)	0.0286 (8)	0.0023 (6)	−0.0031 (6)	−0.0006 (6)
N1	0.029 (4)	0.021 (5)	0.035 (6)	0.004 (3)	−0.002 (4)	−0.002 (4)
N2	0.017 (3)	0.025 (4)	0.027 (5)	−0.003 (3)	0.001 (4)	0.000 (4)
N3	0.033 (5)	0.025 (5)	0.034 (5)	0.007 (4)	−0.007 (4)	−0.003 (4)
N4	0.026 (4)	0.037 (5)	0.029 (5)	−0.007 (4)	−0.005 (4)	−0.008 (4)
N5	0.026 (4)	0.040 (6)	0.038 (6)	0.002 (4)	−0.002 (4)	−0.005 (4)
N6	0.025 (4)	0.027 (5)	0.034 (6)	0.002 (4)	−0.005 (4)	0.002 (4)
C1	0.025 (5)	0.027 (6)	0.043 (7)	−0.008 (5)	−0.008 (5)	0.008 (5)
C2	0.043 (7)	0.032 (7)	0.047 (8)	−0.015 (5)	−0.010 (6)	0.008 (6)
C3	0.025 (6)	0.053 (8)	0.042 (8)	−0.002 (5)	0.001 (5)	0.005 (6)
C4	0.023 (5)	0.038 (7)	0.042 (7)	0.003 (5)	0.004 (5)	−0.006 (5)
C5	0.024 (5)	0.027 (6)	0.030 (6)	−0.005 (4)	−0.010 (5)	0.006 (4)
C6	0.023 (5)	0.030 (6)	0.031 (6)	0.000 (4)	0.005 (4)	−0.004 (5)
C7	0.024 (5)	0.030 (6)	0.037 (7)	−0.003 (4)	0.000 (5)	0.002 (5)
C8	0.022 (5)	0.027 (6)	0.029 (6)	−0.002 (4)	0.005 (4)	0.001 (5)
C9	0.036 (6)	0.032 (7)	0.026 (6)	−0.007 (4)	0.000 (5)	−0.002 (5)
C10	0.045 (7)	0.049 (8)	0.025 (7)	−0.012 (6)	−0.005 (5)	−0.010 (5)
C11	0.035 (6)	0.048 (7)	0.035 (7)	−0.007 (5)	−0.012 (6)	0.002 (6)
C12	0.034 (6)	0.040 (7)	0.031 (7)	0.003 (5)	−0.004 (5)	0.004 (5)
C13	0.025 (5)	0.029 (6)	0.028 (6)	0.002 (4)	−0.004 (4)	0.003 (5)
C14	0.052 (7)	0.022 (6)	0.052 (8)	0.005 (5)	−0.020 (6)	0.003 (5)
C15	0.029 (6)	0.054 (8)	0.050 (9)	0.023 (5)	−0.013 (6)	−0.020 (7)
C16	0.030 (6)	0.054 (8)	0.030 (7)	0.009 (5)	−0.005 (5)	−0.015 (7)
C17	0.026 (6)	0.077 (11)	0.044 (8)	0.010 (6)	−0.001 (5)	−0.031 (8)
C18	0.042 (7)	0.075 (11)	0.045 (9)	−0.013 (7)	0.007 (6)	−0.021 (8)
C19	0.037 (6)	0.060 (9)	0.037 (8)	−0.004 (6)	0.007 (5)	−0.015 (6)
C20	0.034 (6)	0.039 (7)	0.030 (6)	−0.004 (5)	0.000 (5)	−0.007 (5)
C21	0.023 (5)	0.044 (7)	0.036 (7)	0.000 (5)	−0.003 (5)	0.008 (5)
C22	0.034 (6)	0.047 (7)	0.043 (7)	−0.001 (5)	−0.004 (5)	−0.018 (6)
C23	0.029 (6)	0.024 (6)	0.035 (7)	−0.003 (4)	−0.003 (5)	−0.002 (5)
C24	0.028 (6)	0.048 (8)	0.055 (9)	0.002 (5)	−0.006 (5)	0.012 (6)

### (S,S)-Bis(acetonitrile-κN)[N,N'-dimethyl-N,N'-bis(pyridin-2-ylmethyl)cyclohexane-1,2-diamine-κ4N]iron(II) bis(hexafluoroantimonate) (I) Geometric parameters (Å, º)

**Table:**

Sb1—F1	1.856 (7)	C7—H7A	0.9800
Sb1—F2	1.870 (7)	C7—H7B	0.9800
Sb1—F3	1.870 (7)	C7—H7C	0.9800
Sb1—F4	1.845 (9)	C8—H8	1.0000
Sb1—F5	1.842 (9)	C8—C9	1.528 (14)
Sb1—F6	1.820 (10)	C8—C13	1.534 (13)
Sb2—F7	1.884 (7)	C9—H9A	0.9900
Sb2—F8	1.876 (6)	C9—H9B	0.9900
Sb2—F9	1.859 (8)	C9—C10	1.515 (15)
Sb2—F10	1.874 (7)	C10—H10A	0.9900
Sb2—F11	1.866 (7)	C10—H10B	0.9900
Sb2—F12	1.854 (7)	C10—C11	1.553 (16)
Fe1—N1	1.995 (9)	C11—H11A	0.9900
Fe1—N2	2.042 (8)	C11—H11B	0.9900
Fe1—N3	2.031 (9)	C11—C12	1.525 (15)
Fe1—N4	1.985 (9)	C12—H12A	0.9900
Fe1—N5	1.942 (9)	C12—H12B	0.9900
Fe1—N6	1.950 (9)	C12—C13	1.537 (14)
N1—C1	1.350 (12)	C13—H13	1.0000
N1—C5	1.352 (13)	C14—H14A	0.9800
N2—C6	1.484 (11)	C14—H14B	0.9800
N2—C7	1.480 (12)	C14—H14C	0.9800
N2—C8	1.513 (13)	C15—H15A	0.9900
N3—C13	1.504 (13)	C15—H15B	0.9900
N3—C14	1.482 (14)	C15—C16	1.467 (18)
N3—C15	1.495 (14)	C16—C17	1.397 (16)
N4—C16	1.344 (14)	C17—H17	0.9500
N4—C20	1.342 (14)	C17—C18	1.39 (2)
N5—C21	1.148 (13)	C18—H18	0.9500
N6—C23	1.118 (13)	C18—C19	1.393 (18)
C1—H1	0.9500	C19—H19	0.9500
C1—C2	1.375 (15)	C19—C20	1.389 (15)
C2—H2	0.9500	C20—H20	0.9500
C2—C3	1.401 (16)	C21—C22	1.459 (15)
C3—H3	0.9500	C22—H22A	0.9800
C3—C4	1.387 (16)	C22—H22B	0.9800
C4—H4	0.9500	C22—H22C	0.9800
C4—C5	1.388 (14)	C23—C24	1.484 (15)
C5—C6	1.495 (14)	C24—H24A	0.9800
C6—H6A	0.9900	C24—H24B	0.9800
C6—H6B	0.9900	C24—H24C	0.9800
			
F1—Sb1—F2	90.0 (4)	H6A—C6—H6B	108.2
F1—Sb1—F3	178.2 (5)	N2—C7—H7A	109.5
F3—Sb1—F2	88.6 (4)	N2—C7—H7B	109.5
F4—Sb1—F1	90.5 (5)	N2—C7—H7C	109.5
F4—Sb1—F2	87.1 (4)	H7A—C7—H7B	109.5
F4—Sb1—F3	90.6 (5)	H7A—C7—H7C	109.5
F5—Sb1—F1	87.8 (4)	H7B—C7—H7C	109.5
F5—Sb1—F2	177.7 (4)	N2—C8—H8	107.3
F5—Sb1—F3	93.6 (4)	N2—C8—C9	115.5 (8)
F5—Sb1—F4	93.5 (5)	N2—C8—C13	108.2 (8)
F6—Sb1—F1	89.6 (5)	C9—C8—H8	107.3
F6—Sb1—F2	89.1 (4)	C9—C8—C13	110.9 (8)
F6—Sb1—F3	89.2 (5)	C13—C8—H8	107.3
F6—Sb1—F4	176.2 (5)	C8—C9—H9A	109.3
F6—Sb1—F5	90.3 (6)	C8—C9—H9B	109.3
F8—Sb2—F7	91.4 (3)	H9A—C9—H9B	108.0
F9—Sb2—F7	90.1 (4)	C10—C9—C8	111.4 (9)
F9—Sb2—F8	89.4 (4)	C10—C9—H9A	109.3
F9—Sb2—F10	90.0 (4)	C10—C9—H9B	109.3
F9—Sb2—F11	92.7 (5)	C9—C10—H10A	109.9
F10—Sb2—F7	179.0 (3)	C9—C10—H10B	109.9
F10—Sb2—F8	89.6 (3)	C9—C10—C11	109.1 (9)
F11—Sb2—F7	89.7 (3)	H10A—C10—H10B	108.3
F11—Sb2—F8	177.7 (4)	C11—C10—H10A	109.9
F11—Sb2—F10	89.3 (3)	C11—C10—H10B	109.9
F12—Sb2—F7	89.7 (4)	C10—C11—H11A	109.8
F12—Sb2—F8	88.5 (4)	C10—C11—H11B	109.8
F12—Sb2—F9	177.9 (4)	H11A—C11—H11B	108.3
F12—Sb2—F10	90.3 (4)	C12—C11—C10	109.3 (9)
F12—Sb2—F11	89.4 (4)	C12—C11—H11A	109.8
N1—Fe1—N2	81.5 (3)	C12—C11—H11B	109.8
N1—Fe1—N3	96.9 (3)	C11—C12—H12A	109.5
N3—Fe1—N2	85.9 (3)	C11—C12—H12B	109.5
N4—Fe1—N1	178.5 (4)	C11—C12—C13	110.6 (9)
N4—Fe1—N2	98.0 (3)	H12A—C12—H12B	108.1
N4—Fe1—N3	81.7 (4)	C13—C12—H12A	109.5
N5—Fe1—N1	93.7 (4)	C13—C12—H12B	109.5
N5—Fe1—N2	175.1 (4)	N3—C13—C8	108.5 (8)
N5—Fe1—N3	94.0 (4)	N3—C13—C12	115.1 (9)
N5—Fe1—N4	86.7 (4)	N3—C13—H13	107.1
N5—Fe1—N6	88.8 (4)	C8—C13—C12	111.5 (9)
N6—Fe1—N1	87.0 (4)	C8—C13—H13	107.1
N6—Fe1—N2	91.7 (3)	C12—C13—H13	107.1
N6—Fe1—N3	175.1 (4)	N3—C14—H14A	109.5
N6—Fe1—N4	94.5 (4)	N3—C14—H14B	109.5
C1—N1—Fe1	127.1 (7)	N3—C14—H14C	109.5
C1—N1—C5	118.3 (9)	H14A—C14—H14B	109.5
C5—N1—Fe1	114.4 (7)	H14A—C14—H14C	109.5
C6—N2—Fe1	106.6 (6)	H14B—C14—H14C	109.5
C6—N2—C8	107.8 (8)	N3—C15—H15A	109.8
C7—N2—Fe1	113.4 (6)	N3—C15—H15B	109.8
C7—N2—C6	108.3 (8)	H15A—C15—H15B	108.2
C7—N2—C8	112.4 (7)	C16—C15—N3	109.5 (9)
C8—N2—Fe1	108.1 (6)	C16—C15—H15A	109.8
C13—N3—Fe1	108.0 (6)	C16—C15—H15B	109.8
C14—N3—Fe1	114.4 (7)	N4—C16—C15	115.3 (10)
C14—N3—C13	112.3 (9)	N4—C16—C17	121.5 (12)
C14—N3—C15	109.3 (9)	C17—C16—C15	123.2 (11)
C15—N3—Fe1	105.3 (7)	C16—C17—H17	120.2
C15—N3—C13	107.1 (8)	C18—C17—C16	119.5 (12)
C16—N4—Fe1	114.0 (8)	C18—C17—H17	120.2
C20—N4—Fe1	126.8 (8)	C17—C18—H18	120.8
C20—N4—C16	119.2 (10)	C17—C18—C19	118.4 (12)
C21—N5—Fe1	169.9 (9)	C19—C18—H18	120.8
C23—N6—Fe1	173.2 (9)	C18—C19—H19	120.5
N1—C1—H1	118.9	C20—C19—C18	119.0 (13)
N1—C1—C2	122.2 (11)	C20—C19—H19	120.5
C2—C1—H1	118.9	N4—C20—C19	122.3 (11)
C1—C2—H2	120.1	N4—C20—H20	118.9
C1—C2—C3	119.8 (10)	C19—C20—H20	118.9
C3—C2—H2	120.1	N5—C21—C22	179.1 (12)
C2—C3—H3	121.1	C21—C22—H22A	109.5
C4—C3—C2	117.9 (10)	C21—C22—H22B	109.5
C4—C3—H3	121.1	C21—C22—H22C	109.5
C3—C4—H4	120.3	H22A—C22—H22B	109.5
C3—C4—C5	119.4 (10)	H22A—C22—H22C	109.5
C5—C4—H4	120.3	H22B—C22—H22C	109.5
N1—C5—C4	122.3 (9)	N6—C23—C24	177.4 (12)
N1—C5—C6	115.0 (9)	C23—C24—H24A	109.5
C4—C5—C6	122.6 (10)	C23—C24—H24B	109.5
N2—C6—C5	109.8 (8)	C23—C24—H24C	109.5
N2—C6—H6A	109.7	H24A—C24—H24B	109.5
N2—C6—H6B	109.7	H24A—C24—H24C	109.5
C5—C6—H6A	109.7	H24B—C24—H24C	109.5
C5—C6—H6B	109.7		
			
Fe1—N1—C1—C2	−175.3 (8)	C6—N2—C8—C9	−83.2 (10)
Fe1—N1—C5—C4	176.2 (8)	C6—N2—C8—C13	151.8 (8)
Fe1—N1—C5—C6	−1.4 (11)	C7—N2—C6—C5	159.4 (9)
Fe1—N2—C6—C5	37.1 (9)	C7—N2—C8—C9	36.0 (11)
Fe1—N2—C8—C9	161.9 (7)	C7—N2—C8—C13	−88.9 (9)
Fe1—N2—C8—C13	36.9 (8)	C8—N2—C6—C5	−78.7 (9)
Fe1—N3—C13—C8	39.8 (10)	C8—C9—C10—C11	59.5 (12)
Fe1—N3—C13—C12	165.5 (7)	C9—C8—C13—N3	−178.9 (8)
Fe1—N3—C15—C16	39.4 (10)	C9—C8—C13—C12	53.3 (12)
Fe1—N4—C16—C15	1.5 (13)	C9—C10—C11—C12	−60.7 (13)
Fe1—N4—C16—C17	179.8 (9)	C10—C11—C12—C13	58.8 (12)
Fe1—N4—C20—C19	−178.3 (8)	C11—C12—C13—N3	−179.7 (9)
N1—C1—C2—C3	−0.2 (16)	C11—C12—C13—C8	−55.6 (12)
N1—C5—C6—N2	−24.6 (12)	C13—N3—C15—C16	−75.3 (11)
N2—C8—C9—C10	−179.7 (8)	C13—C8—C9—C10	−56.0 (12)
N2—C8—C13—N3	−51.2 (10)	C14—N3—C13—C8	−87.3 (10)
N2—C8—C13—C12	−179.0 (8)	C14—N3—C13—C12	38.4 (12)
N3—C15—C16—N4	−28.2 (14)	C14—N3—C15—C16	162.8 (9)
N3—C15—C16—C17	153.5 (11)	C15—N3—C13—C8	152.7 (9)
N4—C16—C17—C18	−0.7 (18)	C15—N3—C13—C12	−81.6 (11)
C1—N1—C5—C4	1.4 (15)	C15—C16—C17—C18	177.4 (12)
C1—N1—C5—C6	−176.2 (9)	C16—N4—C20—C19	−0.5 (16)
C1—C2—C3—C4	1.4 (17)	C16—C17—C18—C19	−1.5 (19)
C2—C3—C4—C5	−1.3 (17)	C17—C18—C19—C20	2.7 (19)
C3—C4—C5—N1	−0.2 (16)	C18—C19—C20—N4	−1.7 (17)
C3—C4—C5—C6	177.3 (10)	C20—N4—C16—C15	−176.5 (10)
C4—C5—C6—N2	157.8 (10)	C20—N4—C16—C17	1.8 (16)
C5—N1—C1—C2	−1.3 (15)		

### (S,S)-Bis(acetonitrile-κN)[N,N'-dimethyl-N,N'-bis(pyridin-2-ylmethyl)cyclohexane-1,2-diamine-κ4N]iron(II) bis(hexafluoroantimonate) (I) Hydrogen-bond geometry (Å, º)

**Table:**

*D*—H···*A*	*D*—H	H···*A*	*D*···*A*	*D*—H···*A*
C6—H6*A*···F3^i^	0.99	2.45	3.141 (13)	127
C6—H6*B*···F12^ii^	0.99	2.38	3.302 (13)	154
C7—H7*A*···F1	0.98	2.39	3.307 (13)	157
C17—H17···F9^iii^	0.95	2.41	3.260 (15)	149
C19—H19···F3^iv^	0.95	2.52	3.372 (17)	150
C20—H20···F4^iv^	0.95	2.54	3.385 (15)	148
C22—H22*A*···F6^v^	0.98	2.51	3.113 (16)	120
C24—H24*B*···F1^iv^	0.98	2.44	3.232 (16)	138

Symmetry codes: (i) *x*−1, *y*, *z*; (ii) −*x*+1/2, −*y*+1, *z*+1/2; (iii) *x*+1, *y*, *z*; (iv) *x*−1/2, −*y*+3/2, −*z*+1; (v) −*x*+3/2, −*y*+1, *z*−1/2.

### (R,R)-Bis(acetonitrile-κN)[N,N'-dimethyl-N,N'-bis(pyridin-2-ylmethyl)cyclohexane-1,2-diamine-κ4N]iron(II) bis(hexafluoroantimonate) (II) Crystal data

**Table:**

[Fe(C_22_H_28_N_4_)(C_2_H_3_N)_2_](SbF_6_)_2_	*D*_x_ = 1.918 Mg m^−^^3^
*M**_r_* = 933.92	Mo *K*α radiation, λ = 0.71073 Å
Orthorhombic, *P*2_1_2_1_2_1_	Cell parameters from 4610 reflections
*a* = 11.4986 (4) Å	θ = 2.2–22.2°
*b* = 15.1454 (4) Å	µ = 2.20 mm^−^^1^
*c* = 18.5733 (5) Å	*T* = 173 K
*V* = 3234.56 (17) Å^3^	Block, red
*Z* = 4	0.15 × 0.13 × 0.08 mm
*F*(000) = 1824	

### (R,R)-Bis(acetonitrile-κN)[N,N'-dimethyl-N,N'-bis(pyridin-2-ylmethyl)cyclohexane-1,2-diamine-κ4N]iron(II) bis(hexafluoroantimonate) (II) Data collection

**Table:**

Bruker APEXII CCD diffractometer	6490 reflections with *I* > 2σ(*I*)
φ and ω scans	*R*_int_ = 0.095
Absorption correction: multi-scan (SADABS; Krause *et al.*, 2015)	θ_max_ = 30.0°, θ_min_ = 1.7°
*T*_min_ = 0.582, *T*_max_ = 0.746	*h* = −15→16
40783 measured reflections	*k* = −21→21
9446 independent reflections	*l* = −25→26

### (R,R)-Bis(acetonitrile-κN)[N,N'-dimethyl-N,N'-bis(pyridin-2-ylmethyl)cyclohexane-1,2-diamine-κ4N]iron(II) bis(hexafluoroantimonate) (II) Refinement

**Table:**

Refinement on *F*^2^	Hydrogen site location: inferred from neighbouring sites
Least-squares matrix: full	H-atom parameters constrained
*R*[*F*^2^ > 2σ(*F*^2^)] = 0.064	*w* = 1/[σ^2^(*F*_o_^2^) + (0.0279*P*)^2^ + 4.3489*P*] where *P* = (*F*_o_^2^ + 2*F*_c_^2^)/3
*wR*(*F*^2^) = 0.110	(Δ/σ)_max_ = 0.001
*S* = 1.05	Δρ_max_ = 1.36 e Å^−^^3^
9446 reflections	Δρ_min_ = −1.25 e Å^−^^3^
410 parameters	Absolute structure: Flack *x* determined using 2001 quotients [(*I*^+^)-(*I*^-^)]/[(*I*^+^)+(*I*^-^)] (Parsons *et al.*, 2013)
0 restraints	Absolute structure parameter: −0.021 (17)
Primary atom site location: dual	

### (R,R)-Bis(acetonitrile-κN)[N,N'-dimethyl-N,N'-bis(pyridin-2-ylmethyl)cyclohexane-1,2-diamine-κ4N]iron(II) bis(hexafluoroantimonate) (II) Special details

**Table:**

Geometry. All esds (except the esd in the dihedral angle between two l.s. planes) are estimated using the full covariance matrix. The cell esds are taken into account individually in the estimation of esds in distances, angles and torsion angles; correlations between esds in cell parameters are only used when they are defined by crystal symmetry. An approximate (isotropic) treatment of cell esds is used for estimating esds involving l.s. planes.

### (R,R)-Bis(acetonitrile-κN)[N,N'-dimethyl-N,N'-bis(pyridin-2-ylmethyl)cyclohexane-1,2-diamine-κ4N]iron(II) bis(hexafluoroantimonate) (II) Fractional atomic coordinates and isotropic or equivalent isotropic displacement parameters (Å^2^)

**Table:**

	*x*	*y*	*z*	*U*_iso_*/*U*_eq_	
Sb1	1.02104 (6)	0.72826 (5)	0.33421 (4)	0.04228 (18)	
F1	1.0172 (6)	0.8500 (4)	0.3486 (3)	0.0587 (17)	
F2	1.1818 (6)	0.7338 (6)	0.3312 (6)	0.109 (3)	
F3	1.0132 (10)	0.7479 (7)	0.2365 (4)	0.126 (4)	
F4	1.0189 (8)	0.6078 (5)	0.3200 (6)	0.118 (3)	
F5	0.8609 (5)	0.7245 (5)	0.3368 (5)	0.089 (3)	
F6	1.0272 (8)	0.7169 (5)	0.4335 (4)	0.095 (3)	
Sb2	0.16566 (6)	0.38109 (5)	0.56553 (4)	0.04000 (17)	
F7	0.3088 (5)	0.4372 (4)	0.5814 (4)	0.0533 (16)	
F8	0.1456 (7)	0.4483 (6)	0.4823 (4)	0.084 (3)	
F9	0.2370 (5)	0.2916 (4)	0.5125 (3)	0.0587 (18)	
F10	0.0230 (6)	0.3255 (5)	0.5541 (4)	0.086 (3)	
F11	0.1904 (7)	0.3170 (4)	0.6492 (3)	0.067 (2)	
F12	0.0920 (5)	0.4701 (4)	0.6189 (3)	0.0537 (16)	
Fe1	0.53484 (11)	0.50741 (8)	0.40270 (6)	0.0248 (3)	
N1	0.6758 (6)	0.5454 (5)	0.4559 (4)	0.0280 (16)	
N2	0.6562 (6)	0.4505 (4)	0.3388 (4)	0.0287 (16)	
N3	0.5319 (6)	0.6063 (4)	0.3288 (4)	0.0232 (14)	
N4	0.3956 (6)	0.4684 (4)	0.3480 (4)	0.0260 (16)	
N5	0.4284 (6)	0.5712 (5)	0.4650 (4)	0.0273 (16)	
N6	0.5268 (7)	0.4088 (5)	0.4689 (4)	0.0339 (17)	
C1	0.6829 (8)	0.6085 (6)	0.5061 (5)	0.034 (2)	
H1	0.614713	0.640592	0.518279	0.041*	
C2	0.7865 (9)	0.6290 (8)	0.5414 (5)	0.044 (3)	
H2	0.788704	0.673998	0.576994	0.053*	
C3	0.8858 (10)	0.5827 (8)	0.5238 (6)	0.053 (3)	
H3	0.957531	0.595238	0.546975	0.063*	
C4	0.8790 (9)	0.5175 (8)	0.4716 (6)	0.048 (3)	
H4	0.946432	0.485470	0.457738	0.058*	
C5	0.7731 (7)	0.4997 (7)	0.4401 (5)	0.034 (2)	
C6	0.7552 (9)	0.4261 (6)	0.3868 (5)	0.039 (2)	
H6A	0.826629	0.417234	0.357971	0.047*	
H6B	0.737601	0.370391	0.412548	0.047*	
C7	0.6156 (9)	0.3693 (6)	0.3005 (5)	0.040 (2)	
H7A	0.580941	0.328300	0.335208	0.061*	
H7B	0.681938	0.340763	0.276803	0.061*	
H7C	0.557493	0.385490	0.264225	0.061*	
C8	0.6992 (7)	0.5188 (6)	0.2859 (5)	0.0274 (19)	
H8	0.753019	0.558491	0.313148	0.033*	
C9	0.7675 (8)	0.4837 (6)	0.2221 (5)	0.035 (2)	
H9A	0.718670	0.441576	0.194795	0.042*	
H9B	0.836902	0.451534	0.239689	0.042*	
C10	0.8058 (8)	0.5583 (6)	0.1725 (5)	0.039 (2)	
H10A	0.847939	0.533509	0.130685	0.046*	
H10B	0.859419	0.598095	0.198686	0.046*	
C11	0.7005 (8)	0.6106 (7)	0.1463 (5)	0.037 (2)	
H11A	0.648800	0.571666	0.117871	0.044*	
H11B	0.726521	0.659518	0.114827	0.044*	
C12	0.6340 (7)	0.6479 (6)	0.2107 (5)	0.030 (2)	
H12A	0.683908	0.690822	0.236423	0.036*	
H12B	0.564148	0.679574	0.193383	0.036*	
C13	0.5971 (7)	0.5751 (6)	0.2628 (5)	0.0263 (19)	
H13	0.542991	0.535511	0.235675	0.032*	
C14	0.5773 (7)	0.6922 (5)	0.3560 (5)	0.029 (2)	
H14A	0.540290	0.706193	0.402158	0.043*	
H14B	0.559717	0.738839	0.321097	0.043*	
H14C	0.661645	0.688049	0.362653	0.043*	
C15	0.4075 (6)	0.6192 (6)	0.3092 (5)	0.0267 (18)	
H15A	0.402186	0.648993	0.261899	0.032*	
H15B	0.369332	0.657374	0.345524	0.032*	
C16	0.3463 (7)	0.5314 (5)	0.3059 (5)	0.0281 (19)	
C17	0.2470 (8)	0.5188 (7)	0.2673 (5)	0.037 (2)	
H17	0.215783	0.565061	0.238685	0.044*	
C18	0.1918 (9)	0.4370 (6)	0.2703 (5)	0.040 (2)	
H18	0.123244	0.425733	0.243300	0.048*	
C19	0.2403 (9)	0.3723 (7)	0.3141 (5)	0.042 (3)	
H19	0.204262	0.316020	0.317794	0.051*	
C20	0.3395 (8)	0.3893 (6)	0.3519 (5)	0.033 (2)	
H20	0.370685	0.344389	0.381905	0.040*	
C21	0.3574 (8)	0.6013 (5)	0.4996 (5)	0.028 (2)	
C22	0.2631 (8)	0.6411 (6)	0.5417 (5)	0.041 (3)	
H22A	0.190802	0.639301	0.513779	0.062*	
H22B	0.282678	0.702552	0.552876	0.062*	
H22C	0.252707	0.608023	0.586613	0.062*	
C23	0.5163 (8)	0.3580 (6)	0.5148 (5)	0.033 (2)	
C24	0.5025 (8)	0.2951 (6)	0.5737 (5)	0.038 (2)	
H24A	0.437606	0.255272	0.563046	0.057*	
H24B	0.486487	0.327155	0.618475	0.057*	
H24C	0.574134	0.260715	0.579176	0.057*	

### (R,R)-Bis(acetonitrile-κN)[N,N'-dimethyl-N,N'-bis(pyridin-2-ylmethyl)cyclohexane-1,2-diamine-κ4N]iron(II) bis(hexafluoroantimonate) (II) Atomic displacement parameters (Å^2^)

**Table:**

	*U*^11^	*U*^22^	*U*^33^	*U*^12^	*U*^13^	*U*^23^
Sb1	0.0286 (3)	0.0521 (4)	0.0461 (4)	0.0048 (3)	−0.0021 (3)	−0.0136 (3)
F1	0.065 (4)	0.049 (3)	0.063 (4)	0.004 (3)	−0.010 (3)	0.001 (3)
F2	0.028 (4)	0.102 (6)	0.196 (9)	0.017 (4)	0.000 (5)	−0.047 (7)
F3	0.155 (9)	0.183 (9)	0.039 (4)	−0.027 (8)	0.008 (5)	−0.033 (5)
F4	0.091 (6)	0.060 (5)	0.204 (10)	0.016 (5)	−0.031 (7)	−0.054 (6)
F5	0.033 (3)	0.102 (6)	0.131 (7)	−0.010 (4)	−0.001 (4)	−0.060 (6)
F6	0.142 (7)	0.084 (5)	0.058 (4)	−0.048 (5)	−0.023 (5)	0.031 (4)
Sb2	0.0317 (3)	0.0489 (4)	0.0393 (4)	0.0009 (3)	0.0093 (3)	0.0007 (3)
F7	0.037 (3)	0.045 (3)	0.079 (5)	−0.007 (3)	0.010 (3)	0.004 (3)
F8	0.073 (5)	0.129 (7)	0.050 (4)	0.043 (5)	0.001 (4)	0.024 (4)
F9	0.044 (4)	0.079 (5)	0.053 (4)	0.007 (3)	0.006 (3)	−0.021 (4)
F10	0.035 (3)	0.110 (5)	0.112 (6)	−0.020 (4)	0.024 (4)	−0.068 (5)
F11	0.114 (6)	0.038 (3)	0.048 (4)	−0.001 (4)	0.015 (4)	0.010 (3)
F12	0.045 (4)	0.053 (4)	0.064 (4)	0.004 (3)	0.014 (3)	−0.007 (3)
Fe1	0.0232 (6)	0.0260 (6)	0.0252 (6)	0.0022 (5)	0.0027 (5)	0.0013 (5)
N1	0.026 (4)	0.037 (4)	0.021 (4)	−0.001 (3)	0.004 (3)	0.008 (3)
N2	0.029 (4)	0.027 (4)	0.030 (4)	0.006 (3)	0.001 (3)	0.005 (3)
N3	0.020 (3)	0.025 (3)	0.025 (3)	0.000 (3)	0.002 (3)	−0.004 (3)
N4	0.025 (4)	0.023 (4)	0.030 (4)	0.002 (3)	0.011 (3)	−0.003 (3)
N5	0.026 (4)	0.033 (4)	0.023 (4)	−0.003 (3)	0.001 (3)	0.004 (3)
N6	0.035 (4)	0.038 (4)	0.029 (4)	0.001 (4)	0.007 (4)	0.005 (3)
C1	0.036 (5)	0.040 (5)	0.027 (5)	−0.009 (4)	0.001 (4)	0.005 (4)
C2	0.043 (6)	0.059 (7)	0.030 (5)	−0.013 (5)	−0.012 (4)	0.011 (5)
C3	0.048 (7)	0.070 (8)	0.040 (7)	−0.012 (6)	−0.017 (5)	0.025 (6)
C4	0.032 (5)	0.067 (8)	0.044 (7)	0.009 (5)	0.005 (5)	0.031 (6)
C5	0.023 (4)	0.052 (6)	0.026 (5)	0.000 (4)	0.001 (4)	0.015 (5)
C6	0.040 (5)	0.040 (5)	0.038 (6)	0.016 (4)	0.008 (4)	0.016 (5)
C7	0.052 (6)	0.025 (5)	0.043 (6)	0.007 (4)	0.019 (5)	−0.007 (4)
C8	0.026 (4)	0.031 (5)	0.025 (5)	−0.002 (4)	0.003 (4)	0.001 (4)
C9	0.032 (5)	0.042 (6)	0.029 (5)	0.000 (4)	0.001 (4)	0.002 (4)
C10	0.031 (5)	0.051 (6)	0.034 (5)	−0.005 (4)	0.011 (4)	0.003 (5)
C11	0.041 (5)	0.043 (6)	0.026 (5)	−0.006 (5)	−0.003 (4)	0.006 (4)
C12	0.030 (5)	0.032 (5)	0.028 (5)	−0.001 (4)	−0.003 (4)	0.003 (4)
C13	0.021 (4)	0.032 (5)	0.026 (5)	−0.005 (3)	−0.003 (4)	0.003 (4)
C14	0.027 (4)	0.025 (4)	0.035 (5)	−0.005 (3)	−0.004 (4)	−0.003 (4)
C15	0.016 (4)	0.030 (4)	0.034 (5)	0.001 (4)	−0.003 (3)	0.002 (4)
C16	0.023 (4)	0.028 (4)	0.034 (5)	0.001 (4)	0.006 (4)	−0.003 (4)
C17	0.031 (5)	0.041 (6)	0.038 (6)	0.003 (4)	0.005 (4)	0.005 (5)
C18	0.036 (5)	0.043 (6)	0.041 (6)	−0.012 (4)	−0.002 (5)	−0.005 (5)
C19	0.053 (6)	0.035 (5)	0.040 (6)	−0.019 (5)	0.013 (5)	−0.004 (5)
C20	0.039 (5)	0.029 (4)	0.032 (5)	−0.012 (4)	0.010 (4)	−0.003 (4)
C21	0.024 (5)	0.028 (5)	0.032 (5)	−0.003 (4)	−0.003 (4)	0.005 (4)
C22	0.031 (5)	0.044 (6)	0.049 (6)	0.008 (4)	0.013 (4)	−0.015 (5)
C23	0.027 (4)	0.037 (5)	0.035 (5)	0.000 (4)	0.005 (4)	−0.004 (4)
C24	0.043 (6)	0.041 (5)	0.030 (5)	−0.005 (4)	−0.001 (4)	0.010 (4)

### (R,R)-Bis(acetonitrile-κN)[N,N'-dimethyl-N,N'-bis(pyridin-2-ylmethyl)cyclohexane-1,2-diamine-κ4N]iron(II) bis(hexafluoroantimonate) (II) Geometric parameters (Å, º)

**Table:**

Sb1—F1	1.863 (6)	C7—H7A	0.9800
Sb1—F2	1.851 (7)	C7—H7B	0.9800
Sb1—F3	1.842 (8)	C7—H7C	0.9800
Sb1—F4	1.844 (7)	C8—H8	1.0000
Sb1—F5	1.843 (6)	C8—C9	1.518 (12)
Sb1—F6	1.854 (7)	C8—C13	1.513 (12)
Sb2—F7	1.876 (6)	C9—H9A	0.9900
Sb2—F8	1.865 (7)	C9—H9B	0.9900
Sb2—F9	1.866 (6)	C9—C10	1.522 (12)
Sb2—F10	1.856 (6)	C10—H10A	0.9900
Sb2—F11	1.854 (6)	C10—H10B	0.9900
Sb2—F12	1.875 (6)	C10—C11	1.526 (13)
Fe1—N1	1.983 (7)	C11—H11A	0.9900
Fe1—N2	2.024 (7)	C11—H11B	0.9900
Fe1—N3	2.031 (7)	C11—C12	1.528 (12)
Fe1—N4	1.985 (7)	C12—H12A	0.9900
Fe1—N5	1.942 (7)	C12—H12B	0.9900
Fe1—N6	1.936 (7)	C12—C13	1.526 (11)
N1—C1	1.339 (11)	C13—H13	1.0000
N1—C5	1.347 (11)	C14—H14A	0.9800
N2—C6	1.493 (12)	C14—H14B	0.9800
N2—C7	1.496 (11)	C14—H14C	0.9800
N2—C8	1.511 (11)	C15—H15A	0.9900
N3—C13	1.513 (11)	C15—H15B	0.9900
N3—C14	1.490 (10)	C15—C16	1.506 (12)
N3—C15	1.489 (9)	C16—C17	1.362 (13)
N4—C16	1.358 (11)	C17—H17	0.9500
N4—C20	1.363 (10)	C17—C18	1.393 (13)
N5—C21	1.134 (11)	C18—H18	0.9500
N6—C23	1.156 (11)	C18—C19	1.391 (14)
C1—H1	0.9500	C19—H19	0.9500
C1—C2	1.395 (13)	C19—C20	1.363 (13)
C2—H2	0.9500	C20—H20	0.9500
C2—C3	1.379 (16)	C21—C22	1.467 (12)
C3—H3	0.9500	C22—H22A	0.9800
C3—C4	1.386 (16)	C22—H22B	0.9800
C4—H4	0.9500	C22—H22C	0.9800
C4—C5	1.378 (13)	C23—C24	1.459 (12)
C5—C6	1.505 (14)	C24—H24A	0.9800
C6—H6A	0.9900	C24—H24B	0.9800
C6—H6B	0.9900	C24—H24C	0.9800
			
F2—Sb1—F1	89.0 (3)	H6A—C6—H6B	108.4
F2—Sb1—F6	89.8 (4)	N2—C7—H7A	109.5
F3—Sb1—F1	88.9 (4)	N2—C7—H7B	109.5
F3—Sb1—F2	90.7 (5)	N2—C7—H7C	109.5
F3—Sb1—F4	91.1 (5)	H7A—C7—H7B	109.5
F3—Sb1—F5	89.0 (5)	H7A—C7—H7C	109.5
F3—Sb1—F6	176.0 (4)	H7B—C7—H7C	109.5
F4—Sb1—F1	177.9 (4)	N2—C8—H8	106.6
F4—Sb1—F2	93.1 (4)	N2—C8—C9	115.9 (7)
F4—Sb1—F6	92.9 (4)	N2—C8—C13	108.5 (7)
F5—Sb1—F1	90.2 (3)	C9—C8—H8	106.6
F5—Sb1—F2	179.1 (4)	C13—C8—H8	106.6
F5—Sb1—F4	87.7 (4)	C13—C8—C9	112.2 (7)
F5—Sb1—F6	90.5 (4)	C8—C9—H9A	109.4
F6—Sb1—F1	87.2 (3)	C8—C9—H9B	109.4
F8—Sb2—F7	89.5 (3)	C8—C9—C10	111.2 (8)
F8—Sb2—F9	90.8 (3)	H9A—C9—H9B	108.0
F8—Sb2—F12	89.4 (3)	C10—C9—H9A	109.4
F9—Sb2—F7	91.5 (3)	C10—C9—H9B	109.4
F9—Sb2—F12	179.2 (3)	C9—C10—H10A	109.6
F10—Sb2—F7	177.5 (3)	C9—C10—H10B	109.6
F10—Sb2—F8	92.5 (4)	C9—C10—C11	110.4 (7)
F10—Sb2—F9	89.9 (3)	H10A—C10—H10B	108.1
F10—Sb2—F12	89.3 (3)	C11—C10—H10A	109.6
F11—Sb2—F7	88.3 (3)	C11—C10—H10B	109.6
F11—Sb2—F8	177.8 (4)	C10—C11—H11A	109.7
F11—Sb2—F9	89.7 (3)	C10—C11—H11B	109.7
F11—Sb2—F10	89.7 (4)	C10—C11—C12	109.8 (7)
F11—Sb2—F12	90.1 (3)	H11A—C11—H11B	108.2
F12—Sb2—F7	89.3 (3)	C12—C11—H11A	109.7
N1—Fe1—N2	81.5 (3)	C12—C11—H11B	109.7
N1—Fe1—N3	97.8 (3)	C11—C12—H12A	109.3
N1—Fe1—N4	178.9 (3)	C11—C12—H12B	109.3
N2—Fe1—N3	86.0 (3)	H12A—C12—H12B	108.0
N4—Fe1—N2	97.4 (3)	C13—C12—C11	111.6 (7)
N4—Fe1—N3	82.0 (3)	C13—C12—H12A	109.3
N5—Fe1—N1	94.2 (3)	C13—C12—H12B	109.3
N5—Fe1—N2	174.7 (3)	N3—C13—C8	109.3 (7)
N5—Fe1—N3	91.4 (3)	N3—C13—C12	115.2 (7)
N5—Fe1—N4	86.8 (3)	N3—C13—H13	106.7
N6—Fe1—N1	86.9 (3)	C8—C13—C12	111.7 (7)
N6—Fe1—N2	94.4 (3)	C8—C13—H13	106.7
N6—Fe1—N3	175.2 (3)	C12—C13—H13	106.7
N6—Fe1—N4	93.3 (3)	N3—C14—H14A	109.5
N6—Fe1—N5	88.6 (3)	N3—C14—H14B	109.5
C1—N1—Fe1	127.2 (6)	N3—C14—H14C	109.5
C1—N1—C5	117.8 (8)	H14A—C14—H14B	109.5
C5—N1—Fe1	114.9 (6)	H14A—C14—H14C	109.5
C6—N2—Fe1	106.3 (6)	H14B—C14—H14C	109.5
C6—N2—C7	108.6 (7)	N3—C15—H15A	109.7
C6—N2—C8	107.9 (7)	N3—C15—H15B	109.7
C7—N2—Fe1	114.5 (5)	N3—C15—C16	110.0 (7)
C7—N2—C8	110.8 (7)	H15A—C15—H15B	108.2
C8—N2—Fe1	108.4 (5)	C16—C15—H15A	109.7
C13—N3—Fe1	108.0 (5)	C16—C15—H15B	109.7
C14—N3—Fe1	114.2 (5)	N4—C16—C15	113.7 (7)
C14—N3—C13	112.0 (6)	N4—C16—C17	123.7 (8)
C15—N3—Fe1	106.1 (5)	C17—C16—C15	122.5 (8)
C15—N3—C13	108.6 (6)	C16—C17—H17	120.5
C15—N3—C14	107.7 (6)	C16—C17—C18	119.1 (9)
C16—N4—Fe1	115.0 (5)	C18—C17—H17	120.5
C16—N4—C20	116.8 (8)	C17—C18—H18	121.1
C20—N4—Fe1	128.0 (6)	C19—C18—C17	117.9 (9)
C21—N5—Fe1	172.3 (7)	C19—C18—H18	121.1
C23—N6—Fe1	170.9 (7)	C18—C19—H19	119.9
N1—C1—H1	118.7	C20—C19—C18	120.2 (9)
N1—C1—C2	122.6 (10)	C20—C19—H19	119.9
C2—C1—H1	118.7	N4—C20—C19	122.4 (9)
C1—C2—H2	120.6	N4—C20—H20	118.8
C3—C2—C1	118.8 (10)	C19—C20—H20	118.8
C3—C2—H2	120.6	N5—C21—C22	177.7 (10)
C2—C3—H3	120.6	C21—C22—H22A	109.5
C2—C3—C4	118.8 (10)	C21—C22—H22B	109.5
C4—C3—H3	120.6	C21—C22—H22C	109.5
C3—C4—H4	120.5	H22A—C22—H22B	109.5
C5—C4—C3	119.1 (10)	H22A—C22—H22C	109.5
C5—C4—H4	120.5	H22B—C22—H22C	109.5
N1—C5—C4	122.8 (10)	N6—C23—C24	179.0 (9)
N1—C5—C6	114.2 (8)	C23—C24—H24A	109.5
C4—C5—C6	123.0 (9)	C23—C24—H24B	109.5
N2—C6—C5	108.2 (7)	C23—C24—H24C	109.5
N2—C6—H6A	110.0	H24A—C24—H24B	109.5
N2—C6—H6B	110.0	H24A—C24—H24C	109.5
C5—C6—H6A	110.0	H24B—C24—H24C	109.5
C5—C6—H6B	110.0		
			
Fe1—N1—C1—C2	178.7 (7)	C6—N2—C8—C9	79.9 (9)
Fe1—N1—C5—C4	179.6 (7)	C6—N2—C8—C13	−152.9 (7)
Fe1—N1—C5—C6	−2.2 (10)	C7—N2—C6—C5	−164.0 (7)
Fe1—N2—C6—C5	−40.3 (8)	C7—N2—C8—C9	−38.9 (10)
Fe1—N2—C8—C9	−165.3 (6)	C7—N2—C8—C13	88.3 (8)
Fe1—N2—C8—C13	−38.1 (8)	C8—N2—C6—C5	75.8 (9)
Fe1—N3—C13—C8	−36.5 (7)	C8—C9—C10—C11	−57.5 (10)
Fe1—N3—C13—C12	−163.2 (6)	C9—C8—C13—N3	178.9 (7)
Fe1—N3—C15—C16	−38.0 (7)	C9—C8—C13—C12	−52.3 (10)
Fe1—N4—C16—C15	−0.2 (9)	C9—C10—C11—C12	58.4 (10)
Fe1—N4—C16—C17	−176.5 (7)	C10—C11—C12—C13	−56.6 (10)
Fe1—N4—C20—C19	176.3 (7)	C11—C12—C13—N3	179.2 (7)
N1—C1—C2—C3	0.2 (14)	C11—C12—C13—C8	53.7 (10)
N1—C5—C6—N2	28.9 (11)	C13—N3—C15—C16	77.9 (8)
N2—C8—C9—C10	179.8 (7)	C13—C8—C9—C10	54.4 (10)
N2—C8—C13—N3	49.6 (9)	C14—N3—C13—C8	90.1 (8)
N2—C8—C13—C12	178.3 (7)	C14—N3—C13—C12	−36.7 (9)
N3—C15—C16—N4	26.2 (10)	C14—N3—C15—C16	−160.7 (7)
N3—C15—C16—C17	−157.4 (8)	C15—N3—C13—C8	−151.2 (7)
N4—C16—C17—C18	−0.3 (14)	C15—N3—C13—C12	82.1 (8)
C1—N1—C5—C4	−2.2 (13)	C15—C16—C17—C18	−176.2 (8)
C1—N1—C5—C6	175.9 (7)	C16—N4—C20—C19	1.6 (12)
C1—C2—C3—C4	0.0 (15)	C16—C17—C18—C19	1.2 (14)
C2—C3—C4—C5	−1.3 (15)	C17—C18—C19—C20	−0.7 (14)
C3—C4—C5—N1	2.5 (15)	C18—C19—C20—N4	−0.7 (14)
C3—C4—C5—C6	−175.5 (9)	C20—N4—C16—C15	175.1 (7)
C4—C5—C6—N2	−153.0 (9)	C20—N4—C16—C17	−1.1 (12)
C5—N1—C1—C2	0.9 (13)		

### (R,R)-Bis(acetonitrile-κN)[N,N'-dimethyl-N,N'-bis(pyridin-2-ylmethyl)cyclohexane-1,2-diamine-κ4N]iron(II) bis(hexafluoroantimonate) (II) Hydrogen-bond geometry (Å, º)

**Table:**

*D*—H···*A*	*D*—H	H···*A*	*D*···*A*	*D*—H···*A*
C1—H1···F6^i^	0.95	2.54	3.385 (12)	148
C2—H2···F2^ii^	0.95	2.52	3.371 (15)	149
C4—H4···F8^iii^	0.95	2.40	3.246 (13)	148
C14—H14*C*···F5	0.98	2.41	3.317 (10)	155
C15—H15*A*···F11^iv^	0.99	2.40	3.322 (11)	154
C15—H15*B*···F2^v^	0.99	2.46	3.149 (11)	126
C22—H22*B*···F5^i^	0.98	2.50	3.241 (12)	133
C24—H24*B*···F3^vi^	0.98	2.47	3.098 (12)	122

Symmetry codes: (i) *x*−1/2, −*y*+3/2, −*z*+1; (ii) −*x*−1, *y*+3/2, −*z*+3/2; (iii) *x*+1, *y*, *z*; (iv) −*x*+1/2, −*y*+1, *z*−1/2; (v) *x*−1, *y*, *z*; (vi) −*x*+3/2, −*y*+1, *z*+1/2.
